# Autoinjectors Preferred for Intramuscular Epinephrine in Anaphylaxis and Allergic Reactions

**DOI:** 10.5811/westjem.2016.8.30505

**Published:** 2016-10-07

**Authors:** Ronna L. Campbell, M. Fernanda Bellolio, Megan S. Motosue, Kharmene L. Sunga, Christine M. Lohse, Maria I. Rudis

**Affiliations:** * Mayo Clinic, Department of Emergency Medicine, Rochester, Minnesota; † Mayo Clinic, Division of Allergic Diseases, Rochester, Minnesota; ‡ Mayo Clinic, Division of Biomedical Statistics and Informatics, Rochester, Minnesota; § Mayo Clinic, Pharmacy Services, Rochester, Minnesota

## Abstract

**Introduction:**

Epinephrine is the treatment of choice for anaphylaxis. We surveyed emergency department (ED) healthcare providers regarding two methods of intramuscular (IM) epinephrine administration (autoinjector and manual injection) for the management of anaphylaxis and allergic reactions and identified provider perceptions and preferred method of medication delivery.

**Methods:**

This observational study adhered to survey reporting guidelines. It was performed through a Web-based survey completed by healthcare providers at an academic ED. The primary outcomes were assessment of provider perceptions and identification of the preferred IM epinephrine administration method by ED healthcare providers.

**Results:**

Of 217 ED healthcare providers invited to participate, 172 (79%) completed the survey. Overall, 82% of respondents preferred the autoinjector method of epinephrine administration. Providers rated the autoinjector method more favorably for time required for training, ease of use, convenience, satisfaction with weight-based dosing, risk of dosing errors, and speed of administration (*p*<0.001 for all comparisons). However, manual injection use was rated more favorably for risk of provider self-injury and patient cost (*p*<0.001 for both comparisons). Three participants (2%) reported a finger stick injury from an epinephrine autoinjector.

**Conclusion:**

ED healthcare providers preferred the autoinjector method of IM epinephrine administration for the management of anaphylaxis or allergic reactions. Epinephrine autoinjector use may reduce barriers to epinephrine administration for the management of anaphylaxis in the ED.

## INTRODUCTION

Anaphylaxis is a serious allergic reaction that frequently involves multiple organ systems, is rapid in onset, and may cause death.^1^ The management of anaphylactic reactions occurs most commonly in the emergency department (ED), placing emergency care providers on the front line of medical intervention for these patients.^2^,^3^ Epinephrine is the treatment of choice for anaphylaxis,^4^ and delayed administration of epinephrine has been associated with increased risk of death.^5^

Much attention has been focused on the need to improve healthcare delivery and reduce preventable adverse events, including medication errors.^6^ A recent review found that medication errors were most common in the prescribing and administering phases and occurred across all patient age spectrums.^7^ Important sources of error, particularly in neonatal and pediatric patients, were physician inexperience and dosing errors (including 10- and 100-fold dosing errors).^7^ Most serious adverse reactions, including fatalities, associated with epinephrine are a result of improper dosages.^8^ The urgent need of epinephrine administration to a patient with anaphylaxis can result in errors at any stage of the medication-use process: medication ordering, dosing, and administration.^9^,^10^

Several factors contribute to the risk of errors. Epinephrine has historically been supplied in 1:1,000 and 1:10,000 formulations. Both formulations are used in the ED, but the low frequency of epinephrine use in a high-stress context can lead to errors in choosing the correct formulation. The use of a ratio (1:1,000 or 1:10,000) as an expression of drug concentration is uncommon, and the conversion of the ratio to milligrams poses an additional cognitive step. This additional calculation can lead to dosing errors of multiple orders of magnitude. Furthermore, epinephrine can be administered through subcutaneous, intramuscular (IM), or intravenous injection, with increasing bioavailability and greater potential toxicity.

Although autoinjector use may reduce the risk of dosing errors, autoinjector epinephrine is more costly.^11^ Furthermore, patient injury^12^ and injury due to inadvertent autoinjector administration of epinephrine into the finger of the person delivering the medication have been reported in both lay people and healthcare providers.^13^,^14^

Many patients with anaphylaxis are not treated with epinephrine in the ED.^3^,^4^,^15^ While the reasons for this remain poorly understood, we believe that the underestimation of the severity of anaphylaxis, lack of familiarity with the dosing of epinephrine, and fear of complications secondary to inappropriate dosing may be contributing factors.

In our institution, epinephrine autoinjectors (EpiPen 0.3 mg and EpiPenJr 0.15 mg; Mylan Specialty, LP) were made available in automated dispensing cabinets in November 2008 for ED use in anaphylaxis treatment. Before this date, only ampules of epinephrine were available, from which epinephrine was drawn for manual IM injection for anaphylaxis or allergic reactions. After the introduction of epinephrine autoinjectors, we had the distinct opportunity to assess healthcare provider satisfaction, perceptions of safety, experiences, and preferred delivery method of IM epinephrine administration. Further, we hypothesized that understanding provider perceptions could provide information that would indicate the method of epinephrine administration associated with fewer barriers to use. Thus, the objective of the study was to examine healthcare providers’ preferences and perceptions about the optimal mode of epinephrine delivery with respect to safety, effectiveness, ease of administration, convenience, and cost for the two methods of epinephrine administration in management of anaphylaxis and allergic reactions.

## METHODS

### Design and Setting

This study adheres to the guidelines for standardized reporting of survey research^16^,^17^ and guidelines for reporting observational studies (Strengthening the Reporting of Observational Studies in Epidemiology [STROBE]).^18^ This study was approved by the Mayo Clinic Survey Research Center. We developed the survey instrument in collaboration with staff at our institution’s survey research center. Input from an expert on survey design was obtained to design the research tool. We incorporated appropriate survey methodology addressing non-random sampling, questionnaire layout, wording of the questions, and piloting. Two emergency physicians on staff, one pharmacist, and one nurse participated in the pilot testing and refinement of the survey, as well as the final survey. All known eligible participants were invited to complete the survey, which was administered by the research survey center to maintain masking to investigators and participant confidentiality. The full survey is included in the Appendix. The survey was distributed to ED healthcare providers between April 28 and June 16, 2011. Our institution is an academic tertiary care and referral center with approximately 73,000 annual ED visits and an admission rate of 30%. Approximately 20% of the ED patients are younger than 18 years.

### Participants

Participants in the study consisted of healthcare providers, including ED pharmacists, emergency medicine (EM) residents, ED physician assistants, ED nurse practitioners, ED nurses, and ED staff physicians who work at the ED of Mayo Clinic Hospital - Rochester, Saint Marys Campus. We compiled a distribution list of email addresses for all ED providers (N=217), and then verified the status of each person using the internal employee directory. A recruitment email was sent to all ED provider staff; all responses were collected anonymously. After the initial invitation, three reminder emails were sent to nonresponders. No respondents contacted the primary investigator about content questions.

### Variables and Measurements

Data collection included participant demographic characteristics and questions regarding the participant’s perceptions of and experiences with use of epinephrine administration through an epinephrine autoinjector or manual injection for patients with allergic reactions or anaphylaxis. The assessment regarding the two injection methods included questions on ease of use, convenience, satisfaction with weight-based epinephrine dosing, risk of dosing errors, cost to patient, speed of administration, and risk of self-injury. Respondents were asked to place their answers as electronic marks on a scale of 0 to 100.

The initial survey was piloted on a small sample of the target population to identify whether respondents understood the questions and instructions and whether the meaning of questions was the same for all respondents. After feedback, we refined the survey and sent it to the final group of participants.

### Data Collection

REDCap (Research Electronic Data Capture),^19^ a secure Web-based research application hosted at Mayo Clinic in Rochester, Minnesota, was used to collect and manage data.

### Statistical Analysis

We did not perform a sample size calculation because a finite number of ED providers could be queried. Frequencies and proportions for categorical variables were used to describe participant characteristics. These characteristics were compared among occupations using Kruskal-Wallis or Fisher exact tests. We summarized responses to the questions asked for both epinephrine autoinjector and manual injection with mean (SD) and median (interquartile range) as appropriate and compared them using Wilcoxon signed rank tests for paired data. Statistical analyses were performed by a statistician using SAS software package version 9.3 (SAS Institute Inc). *P* values <0.05 were considered statistically significant. We performed a subgroup analysis of the providers who reported experience with both autoinjector and manual injection techniques, and a subgroup analysis of nurses only, because they are the provider most likely to administer the medication.

## RESULTS

Of the 217 ED healthcare providers invited to participate, 172 (79%) completed the survey.

### Demographic Characteristics

Demographic characteristics are depicted in Table 1. Of 172 respondents, 53 (31%) were either EM residents or staff physicians, 103 (60%) were nurses, and 15 (9%) were either advanced practice providers or pharmacists. One provider did not report occupation. Overall, 96 respondents (57%) were women, and the majority of respondents had >10 years of clinical practice experience. Among nurses, respondents were more likely to be women (74%); EM residents, ED staff physicians, and ED pharmacists were more likely to be men (74%, 68%, and 86% male respondents, respectively).

### Epinephrine Administration Experiences, Perceptions, and Preferences

Overall, 147 providers (87%) had either recommended, ordered, or administered epinephrine for the management of an allergic reaction or anaphylaxis in the ED. Three providers (2%) reported having a finger stick injury while using an epinephrine autoinjector; all of these respondents were nurses. When asked to estimate the amount of training time required for a provider to safely administer epinephrine, 148 respondents (88%) estimated ≤10 minutes would be adequate for training to safely use an epinephrine autoinjector compared with 94 (57%) who estimated that ≤10 minutes would be adequate for training to safely administer epinephrine with manual IM injection. Overall, 137 respondents (82%) preferred using an epinephrine autoinjector for management of an allergic reaction or anaphylaxis in the ED vs manual IM injection (Table 1).

Providers rated the autoinjector more favorably with regard to ease of use, convenience, satisfaction with weight-based dosing, risk of dosing errors, and speed of administration (*p*<0.001 for all comparisons) (Table 2). However, manual injection was rated more favorably with regard to risk of provider self-injury and patient cost (*p*<0.001 for both comparisons).

### Subgroup Analysis

Some providers did not have ED experience with both methods of epinephrine administration; therefore, we performed a subgroup analysis of the 49 (28.5% of 172 total) providers who reported experience with both autoinjector and manual techniques. This subgroup was similar to the group of unilaterally experienced providers with regard to gender and years of practice (data not shown). Those with experience in both methods were more likely to be ED staff physicians (17/49 [35%] vs 16/123 others [13%], *p*=0.02). The ratings among this subgroup of providers were similar to the overall results except that no significant difference existed between ratings of satisfaction and weight-based dosing (Table 2). We also performed a subgroup analysis of the nurses; the ratings provided by the nurses were similar to the overall group except that there was no significant difference with regard to risk of self-injury.

## DISCUSSION

In this survey of 172 ED healthcare providers, including ED staff physicians, nurses, advanced practice providers, residents, and pharmacists, 82% preferred the autoinjector method of IM epinephrine administration for management of allergic reactions or anaphylaxis. To our knowledge, this is the first study to assess provider preferences with regard to methods of IM administration of epinephrine. ED providers rated the autoinjector method more favorably with regard to amount of time required for training, ease of use, convenience, satisfaction with weight-based dosing, risk of dosing errors, and speed of administration. However, manual injection was rated more favorably with regard to risk of provider self-injury and patient cost.

Importantly, the epinephrine autoinjector was rated much more favorably compared with manual injection for risk of dosing errors. The perceived increase in risk of dosing errors with manual injection may be due to the risks of unfamiliarity with the appropriate dose or route, miscalculation of the dose, and miscommunication between the ordering provider and the nurse administering the medication, as has been suggested previously.^10^ Although anaphylaxis is more commonly managed in the ED than in other clinical settings,^2^,^3^ it is not a common occurrence. A study by Gaeta et al^20^ showed that allergic concerns made up about 1% of ED visits, and only about 1% of these were coded as anaphylaxis. These findings likely underestimate the frequency of anaphylaxis in the ED due to underdiagnosis. More recent data indicate that ED visits for anaphylaxis are increasing.^21^ However, this may be due, at least in part, to increased recognition rather than a true increase in incidence; nevertheless, anaphylaxis continues to be a relatively infrequent emergency in the ED. Its infrequency can lead to unfamiliarity with epinephrine dosing for the ordering provider and the nurse administering the medication and to subsequent increased risk of dosing errors and adverse effects.

Interestingly, although epinephrine autoinjectors are available in only two different doses (0.15 and 0.30 mg), the providers in our study overall rated autoinjectors more favorably with regard to weight-based dosing, whereas the providers who had experience with both methods of IM epinephrine administration rated them similarly. This favorable rating of autoinjectors suggests that providers considered the autoinjector doses, although inexact for weight-based dosing, to be adequate for the majority of patients.

Nevertheless, autoinjectors may not be the best mode of administration for very young patients. In patients weighing <15 kg, autoinjector use could potentially result in overdose, particularly in patients weighing <10 kg. Thus, although the adverse effects of an autoinjector epinephrine dose of 0.15 mg in patients weighing <15 kg are unlikely to be dangerous at the plasma levels achieved,^22^ manual injection may be preferred in this patient population.^23^ Likewise, in patients weighing >30 kg, the autoinjector may result in underdosing of epinephrine. However, manual injection may delay administration because of the time needed to calculate the dose and administer the medication. Finally, studies have found that the autoinjector needle length may be inadequate in a substantial number of children and adults, particularly those with a higher body mass index.^24^,^25^ This inadequate needle length could result in subcutaneous injection rather than IM delivery. Subcutaneous injection has been shown to result in lower peak plasma concentrations than IM administration.^26^,^27^ Conversely, a long needle in children weighing <15 kg may place them at risk of epinephrine being administered into bone.^28^

Although autoinjectors were rated favorably in many respects, overall the providers identified an increased risk of self-injury with the autoinjector. Interestingly, although three nurses reported self-injury with the epinephrine autoinjector, when the nursing responses were analyzed as a subgroup, there was no significant difference in the rating of risk of self-injury. This may be due to the fact that, by the time of the survey, nurses had received additional training to prevent finger stick injuries and therefore did not perceive an increased risk of self-injury. Nonetheless, autoinjector-related finger stick injury has been well documented in the literature and can result in delay in administration.^13^,^14^ Fortunately, death or long-term morbidity have not been reported as related to inadvertent finger self-injection.^29^,^30^ Furthermore, recent reports have documented patient lacerations and embedded needles secondary to autoinjectors.^12^ However, the true incidence of these injuries is unknown and may be mitigated by proper limb immobilization before administration.

Providers also correctly identified that autoinjectors are more expensive than manual injection. As we previously published,^11^ the average wholesale cost of the autoinjectors used in the present study was approximately U.S. $75.00 compared with U.S.$ 3.00 for the 1:1,000 vial of epinephrine. However, more recently, the cost of autoinjectors has increased substantially. Currently, EpiPens are only available in packages of two and have an average wholesale price for the 0.15-mg or 0.3-mg dose of U.S. $730.33, while the average wholesale cost of the 1-mL 1:1,000 vial of epinephrine is U.S. $15.00.^31^ The generic epinephrine autoinjector, Adrenaclick, is sold individually and has an average wholesale price of U.S. $103.50 or as a two-pack for U.S. $206.98.^31^ This cost is substantial and may be a barrier for use of autoinjectors in some EDs. Prefilled epinephrine syringes have been suggested as a potential low-cost alternative to epinephrine autoinjectors and have been shown to be stable and sterile three months after preparation.^32^ Few data exist on the current availability of autoinjectors in EDs or other healthcare settings. One study reported that only one of seven hospitals that responded to a survey reported having epinephrine autoinjectors available in their hospital crash carts.^9^ Furthermore, this cost must be weighed against the potential cost of complications related to delay in epinephrine administration or to epinephrine overdose.

## LIMITATIONS

The present study is limited because only 28% of the respondents had actual ED experience with both epinephrine autoinjectors and manual IM injection of epinephrine. Yet, the perceptions and preferences of the respondents overall were consistent with the respondents who had experience with both methods. Only the EpiPen and EpiPenJr were introduced in our ED, and therefore, perceptions and preferences may have been different if a different brand of autoinjector had been chosen. In addition, although we had an excellent response rate of 79%, the survey may have non-respondent bias because providers most interested in epinephrine administration may have been more likely to respond. Furthermore, there was an approximately 2.5-year period between the introduction of epinephrine autoinjectors and the time of the survey, which could result in recall bias. However, during this time, there was a gradual increase in use of the autoinjectors, and because epinephrine administration for an allergic reaction or anaphylaxis is relatively infrequent, this allowed time for providers to have an opportunity to use the autoinjector. In addition, although autoinjectors were available, providers could continue to use the manual method if they preferred. Finally, the survey was conducted at a single tertiary care ED, and providers in other clinical environments may have different practice patterns, perceptions, and preferences. Thus, larger, multicenter studies should be undertaken to further characterize the risks, benefits, and perceptions of use of autoinjectors vs manually administered epinephrine.

## CONCLUSION

Of ED provider respondents, 82% preferred the autoinjector method of IM epinephrine administration over manual dosing and administration for management of allergic reactions or anaphylaxis. Ultimately, risks and benefits of the two options for IM epinephrine administration must be considered on the basis of the individual patient. Epinephrine autoinjectors, though more costly, provide a rapid and reliable way to administer a life-saving medication in a high-stress situation.^33^,^34^ Thus, autoinjector use may reduce barriers to epinephrine administration for anaphylaxis management in the ED and should be considered to improve patient care.

## Supplementary Information



## Figures and Tables

**Table 1:** Comparison by occupation of responders to survey on use of autoinjector vs. manual injection of epinephrine

Characteristic	Occupations, no. (%)					All (n=172)^a^	*P* value^b^

	ED nurse (n=103)	ED PA/NP (n=7)	EM resident (n=20)	EM staff physician (n=33)	ED pharmacist (n=8)		
Gender (n= 167)							<0.001
Female	76 (74)	3 (50)	5 (26)	10 (32)	1 (14)	96 (57)	
Male	27 (26)	3 (50)	14 (74)	21 (68)	6 (86)	71 (43)	
Years in practice							<0.001
0–3	0	2 (29)	17 (85)	3 (9)	2 (25)	24 (14)	
4–9	26 (25)	4 (57)	3 (15)	11 (33)	5 (63)	49 (28)	
10–20	41 (40)	1 (14)	0	10 (30)	0	52 (30)	
>20	36 (35)	0	0	9 (27)	1 (13)	47 (27)	
Epinephrine recommended, ordered, or administered in ED (n=169)	88 (87)	6 (86)	17 (85)	31 (97)	4 (50)	147 (87)	0.02
Epinephrine formulation used (n=147)^c^							
Autoinjector	71 (81)	3 (50)	17 (100)	21 (68)	4 (100)	116 (79)	0.02
Manual IM injection	36 (41)	1 (17)	3 (18)	21 (68)	1 (25)	62 (42)	0.004
Subcutaneous injection	53 (60)	1 (17)	0	18 (58)	1 (25)	74 (50)	<0.001
IV bolus	24 (27)	3 (50)	3 (18)	11 (35)	0	41 (28)	0.35
IV infusion	17 (19)	0	4 (24)	10 (32)	3 (75)	34 (23)	0.06
Injured using epinephrine autoinjector (n=168)							0.79
No	97 (97)	6 (100)	20 (100)	33 (100)	8 (100)	165 (98)	
Finger stick injury	3 (3)	0	0	0	0	3 (2)	
Other injury	0	0	0	0	0	0	
Injured during manual IM injection (n=168)							NA
No	101 (100)	5 (100)	20 (100)	33 (100)	8 (100)	168 (100)	
Finger stick injury	0	0	0	0	0	0	
Other injury	0	0	0	0	0	0	
Training time for epinephrine autoinjector, min (n=169)							0.66
<5	43 (43)	4 (57)	6 (30)	12 (36)	2 (29)	67 (40)	
5–10	45 (45)	3 (43)	11 (55)	16 (48)	5 (71)	81 (48)	
10–20	10 (10)	0	3 (15)	3 (9)	0	16 (9)	
20–30	2 (2)	0	0	2 (6)	0	4 (2)	
>30	1 (1)	0	0	0	0	1 (1)	
Training time for manual IM injection, min (n=166)							<0.001
<5	23 (23)	1 (14)	0	4 (12)	1 (14)	30 (18)	
5–10	45 (46)	3 (43)	4 (20)	9 (27)	3 (43)	64 (39)	
10–20	25 (26)	3 (43)	9 (45)	9 (27)	3 (43)	49 (30)	
20–30	5 (5)	0	4 (20)	4 (20)	0	17 (10)	
>30	0	0	3 (15)	3 (15)	0	6 (4)	
Overall preference (n=168)							<0.001
Highly prefer autoinjector	72 (73)	2 (29)	17 (85)	14 (42)	4 (50)	109 (65)	
Somewhat prefer autoinjector	13 (13)	2 (29)	3 (15)	6 (18)	3 (38)	28 (17)	
No preference	7 (7)	2 (29)	0	6 (18)	1 (13)	16 (10)	
Somewhat prefer manual IM injection	6 (6)	0	0	6 (18)	0	12 (7)	
Highly prefer manual IM injection	1 (1)	1 (14)	0	1 (3)	0	3 (2)	

*ED,* emergency department; *EM,* emergency medicine; *IM,* intramuscular; *IV,* intravenous; *NA,* not applicable; *NP*, nurse practitioner; *PA,* physician assistant.

a One respondent did not report occupation.

b *P* values for comparisons of features by occupation were obtained with Kruskal-Wallis or Fisher exact tests.

c Respondent could select more than 1 choice.

**Table 2:** Ratings of epinephrine autoinjector and manual intramuscular injection by 172 survey respondents^a^.

Parameter (no. of respondents)^a^	Epinephrine autoinjector,^b^ mean (SD); median (IQR)	Manual IM injection,^b^ mean (SD); median (IQR)	*P* value^c^
Ease of use (161:150)	85.5 (16.4); 90 (80–97)	49.6 (24.7); 50 (29–67)	<0.001
Convenience (162:155)	88.7 (15.0); 94.5 (85–100)	38.2 (26.3); 33 (17–50)	<0.001
Satisfaction with weight-based dosing (152:148)	68.3 (23.5); 69.5 (50–90)	56.7 (25.8); 50 (45–77)	<0.001
Risk of dosing errors (155:152)	20.1 (19.8); 15 (4–27)	67.8 (22.0); 72 (52–83)	<0.001
Cost to patient (133:129)	58.2 (15.9); 50 (50–70)	40.6 (16.9); 50 (27–50)	<0.001
Speed of administration (161:154)	84.1 (16.6); 90 (76–97)	45.7 (23.3); 50 (28–61)	<0.001
Risk of self-injury (155:154)	52.6 (24.8); 58 (30–73)	38.4 (22.4); 39 (20–50)	<0.001
Subset of 49 respondents^d^			
Ease of use (46:44)	87.8 (18.2); 95.5 (85–99)	53.8 (28.9); 50.5 (27–75)	<0.001
Convenience (45:45)	92.5 (10.7); 96 (91–100)	38.3 (29.7); 30 (15–60)	<0.001
Satisfaction with weight-based dosing (47:45)	65.7 (27.2); 60 (50–97)	59.9 (30.7); 65 (40–88)	0.47
Risk of dosing errors (45:45)	19.7 (20.3); 14 (3–26)	71.4 (25.6); 75 (60–94)	<0.001
Cost to patient (41:39)	63.0 (17.5); 61 (50–77)	38.5 (16.9); 50 (25–50)	<0.001
Speed of administration (46:46)	86.2 (16.7); 90.5 (80–98)	44.6 (25.6); 36.5 (25–66)	<0.001
Risk of self-injury (47:47)	57.7 (25.3); 60 (30–79)	36.0 (20.6); 34 (20–50)	<0.001
Subset of 103 ED nurses			
Ease of use (95:90)	87.9 (16.2); 94 (84–99)	50.9 (25.8); 50 (29–67)	<0.001
Convenience (94:92)	89.9 (16.1); 96 (87–100)	39.1 (28.1); 34 (18.5–50)	<0.001
Satisfaction with weight-based dosing (89:87)	70.1 (24.4); 74 (50–94)	54.7 (26.3); 50 (41–75)	<0.001
Risk of dosing errors (92:90)	18.0 (18.7); 11.5 (2.5–26)	68.1 (23.7); 75 (50–86)	<0.001
Cost to patient (73:71)	57.1 (13.8); 50 (50–70)	43.2 (12.6); 50 (36–50)	<0.001
Speed of administration (96:92)	88.0 (14.6); 92 (83.5–98)	46.6 (25.0); 50 (28.5–67.5)	<0.001
Risk of self-injury (91:90)	49.8 (25.0); 50 (26–70)	44.0 (21.7); 50 (26–60)	0.14

*ED,* emergency department; *IM*, intramuscular; *SD,* standard deviation; *IQR,* interquartile range.

a The first number represents the number of respondents who rated the parameter for the epinephrine autoinjector and the second number represents the number of respondents who rated the parameter for manual injection.

b Higher scores indicate increased ease of use, increased convenience, greater satisfaction with weight-based dosing, increased risk of dosing errors, greater cost to patient, higher speed of administration, and higher risk of self-injury scores.

c *P* values obtained from Wilcoxon signed rank tests for paired data.

d Subset of 49 respondents with epinephrine autoinjector and IM manual injection of epinephrine experience in the ED.
