# Supplementary material for: Autoinjectors Preferred for Intramuscular Epinephrine in Anaphylaxis and Allergic Reactions
Source: West J Emerg Med. 2016 Oct 7;17(6):775–82. doi: 10.5811/westjem.2016.8.30505 (PMC5102607; doi:10.5811/westjem.2016.8.30505)
Supplement: Supplementary file 1 [file wjem-17-775-s001.pdf]

Comparison of EpiPen® vs Syringe/Ampule for ED Epinephrine Administration

Epinephrine Ampule	EpiPen
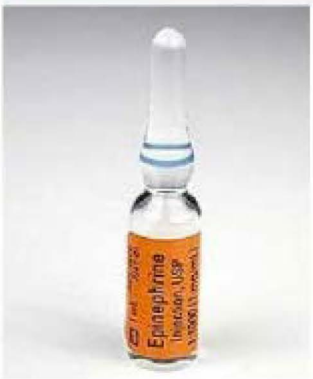	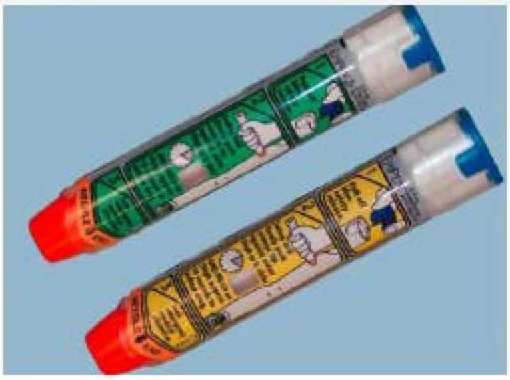

Please answer the following questions.

Have you ever ordered, administered, or recommended epinephrine to treat an allergic reaction or anaphylaxis in the emergency department?

☐ Yes
☐ No

Which formulations have you used? (Check all that apply.)

☐ EpiPen® or EpiPen Jr®
☐ IM epinephrine with syringe
☐ Subcutaneous epinephrine
☐ IV epinephrine BOLUS
☐ IV epinephrine INFUSION

For each of the following questions, compare administration using an EpiPen or EpiPen Jr versus the syringe/ampule (1:1000) method for the treatment of an acute allergic reaction or anaphylaxis in the emergency department. Assume all doses are intramuscular (IM).How EASY is it TO USE each of the following?

EpiPen or EpiPen Jr

Very Difficult — Very Easy

(Place a mark on the scale above)

Syringe/Ampule

Very Difficult — Very Easy

(Place a mark on the scale above)

How CONVENIENT is it to use each of the following?

EpiPen or EpiPen Jr

Very Inconvenient — Very Convenient

(Place a mark on the scale above)

Syringe/Ampule

Very Inconvenient — Very Convenient

(Place a mark on the scale above)

How SATISFIED WITH WEIGHT-BASED DOSING are you with each of the following?

EpiPen or EpiPen Jr

Very Dissatisfied — Very Satisfied

(Place a mark on the scale above)

Syringe/Ampule

Very Dissatisfied — Very Satisfied

(Place a mark on the scale above)

What is the RISK OF DOSING ERRORS with each of the following?

EpiPen or EpiPen Jr

Very Low Risk — Very High Risk

(Place a mark on the scale above)

Syringe/Ampule

Very Low Risk — Very High Risk

(Place a mark on the scale above)

What is the COST TO THE PATIENT for each of the following?

EpiPen or EpiPen Jr

Very Inexpensive — Very Expensive

(Place a mark on the scale above)

Syringe/Ampule

Very Inexpensive — Very Expensive

(Place a mark on the scale above)

What is the SPEED of ADMINISTRATION after the order is placed with each of the following?

EpiPen or EpiPen Jr

Very Slow — Very Fast

(Place a mark on the scale above)

Syringe/Ampule

Very Slow — Very Fast

(Place a mark on the scale above)

What is the RISK OF SERIOUS MEDICATION SIDE EFFECTS (arrhythmia, cardiac ischemia, stroke, other) with each of the following?

EpiPen or EpiPen Jr

Very Low Risk Very High Risk

(Place a mark on the scale above)

Syringe/Ampule

Very Low Risk Very High Risk

(Place a mark on the scale above)

What is the RISK OF SELF INJURY with each of the following?

EpiPen or EpiPen Jr

Very Low Risk Very High Risk

(Place a mark on the scale above)

Syringe/Ampule

Very Low Risk Very High Risk

(Place a mark on the scale above)

What would you estimate TRAINING TIME required for a healthcare provider to safely administer epinephrine for each of the following?

EpiPen or EpiPen Jr

- ☐ Less than 5 minutes
- ☐ 5-10 minutes
- ☐ 10-20 minutes
- ☐ 20-30 minutes
- ☐ More than 30 minutes

Syringe/Ampule

- ☐ Less than 5 minutes
- ☐ 5-10 minutes
- ☐ 10-20 minutes
- ☐ 20-30 minutes
- ☐ More than 30 minutes

When considering all of the differences between the EpiPen or EpiPen Jr and the syringe/ampule method, what is your OVERALL PREFERENCE?

Overall preference

- ☐ Highly prefer EpiPen or EpiPen Jr
- ☐ Somewhat prefer EpiPen or EpiPen Jr
- ☐ No preference
- ☐ Somewhat prefer Syringe/Ampule
- ☐ Highly prefer Syringe/Ampule

For the next questions, consider your personal experiences with administration of epinephrine for anaphylaxis or allergic reactions in the [redacted] ED during approximately the last 3 years.

In the last 3 years, have you personally suffered an injury while administering an EpiPen or EpiPen Jr in the ED?

- ☐ No
- ☐ Yes, finger stick injury
- ☐ Yes, other injury

Please describe injury.

In the last 3 years, have you personally suffered an injury while administering epinephrine via the syringe/ampule method in the ED?

- ☐ No
- ☐ Yes, finger stick injury
- ☐ Yes, other injury

Please describe injury.

In the past 3 years, have you cared for a patient who had a serious ADVERSE SIDE EFFECT from epinephrine (arrhythmia, cardiac ischemia, stroke, other) when you ordered or administered epinephrine for an allergic reaction or anaphylaxis in the ED?

- ☐ No
- ☐ Yes (You will be given the opportunity to answer questions for more than one patient below, if needed.)

For this patient, what was the adverse effect? (Check all that apply.)

- ☐ arrhythmia (other than sinus tachycardia)
- ☐ cardiac ischemia
- ☐ stroke or TIA (transient ischemic attack)
- ☐ other

Please specify the adverse effect.

For this patient, with which formulation of epinephrine did the adverse effect occur?

- ☐ EpiPen or EpiPen Jr
- ☐ IM epinephrine with syringe
- ☐ Subcutaneous epinephrine
- ☐ BOLUS IV epinephrine
- ☐ IV epinephrine INFUSION
- ☐ Unsure

For this patient, was the adverse effect associated with an INCORRECT DOSE of epinephrine?

- ☐ Yes
- ☐ No

For this patient who received the incorrect dose of epinephrine, how did the incorrect dosing occur?

- ☐ Wrong FORMULATION used (1:1000 instead of 1:10,000 or vice versa)
- ☐ Wrong AMOUNT was given (mg or ml)
- ☐ Wrong FORMULATION and AMOUNT
- ☐ Wrong ROUTE was used (Subcutaneous,IM, IV)
- ☐ BOLUS was given instead of INFUSION
- ☐ Other

For this patient, please briefly explain how the incorrect dosing occurred.

Have you cared for any additional patients who had an ADVERSE EFFECT from epinephrine administered in the ED for an allergic reaction or anaphylaxis?

☐ Yes
☐ No

For the second patient, what was the adverse effect? (Check all that apply.)

☐ arrhythmia (other than sinus tachycardia)
☐ cardiac ischemia
☐ stroke or TIA (transient ischemic attack)
☐ other

Please specify the adverse effect.

For the second patient, with which formulation of epinephrine did the adverse effect occur?

☐ EpiPen or EpiPen Jr
☐ IM epinephrine with syringe
☐ Subcutaneous epinephrine
☐ BOLUS IV epinephrine
☐ IV epinephrine INFUSION
☐ Unsure

For the second patient, was the adverse effect associated with an INCORRECT DOSE of epinephrine?

☐ Yes
☐ No

For the second patient, how did the incorrect dosing of epinephrine occur?

☐ Wrong FORMULATION used (1:1000 instead of 1:10,000 or vice versa)
☐ Wrong AMOUNT was given (mg or ml)
☐ Wrong FORMULATION and AMOUNT
☐ Wrong ROUTE was used (Subcutaneous,IM, IV)
☐ BOLUS was given instead of INFUSION
☐ Other

For the second patient, please briefly explain how the incorrect dosing of epinephrine occurred.

Have you cared for any additional patients who had an ADVERSE EFFECT from epinephrine administered in the ED for an allergic reaction or anaphylaxis?

☐ Yes
☐ No

For the third patient, what was the adverse effect? (Check all that apply.)

☐ arrhythmia (other than sinus tachycardia)
☐ cardiac ischemia
☐ stroke or TIA (transient ischemic attack)
☐ other

Please specify the adverse effect.

For the third patient, with which formulation of epinephrine did the adverse effect occur?

- ☐ EpiPen or EpiPen Jr
- ☐ IM epinephrine with syringe
- ☐ Subcutaneous epinephrine
- ☐ BOLUS IV epinephrine
- ☐ IV epinephrine INFUSION
- ☐ Unsure

For the third patient, was the adverse effect associated with an INCORRECT DOSE of epinephrine?

- ☐ Yes
- ☐ No

For the third patient, how did the incorrect dosing of epinephrine occur?

- ☐ Wrong FORMULATION used (1:1000 instead of 1:10,000 or vice versa)
- ☐ Wrong AMOUNT was given (mg or ml)
- ☐ Wrong FORMULATION and AMOUNT
- ☐ Wrong ROUTE was used (Subcutaneous,IM, IV)
- ☐ BOLUS was given instead of INFUSION
- ☐ Other

For the third patient, please briefly explain how the incorrect dosing of epinephrine occurred.

Excluding any patient(s) in question(s) above, in the past 3 years, have you cared for a patient who received an INCORRECT DOSE of epinephrine when you ordered or administered epinephrine for an allergic reaction or anaphylaxis in the ED?

- ☐ No
- ☐ Yes (You will be given the opportunity to answer questions for more than one patient below, if needed.)

With which formulation of epinephrine did the INCORRECT DOSING occur?

- ☐ EpiPen or EpiPen Jr
- ☐ IM epinephrine with syringe
- ☐ Subcutaneous epinephrine
- ☐ BOLUS IV epinephrine
- ☐ IV epinephrine INFUSION
- ☐ Unsure

For your patient who received the incorrect dose of epinephrine, how did the incorrect dosing occur?

- ☐ Wrong FORMULATION used (1:1000 instead of 1:10,000 or vice versa)
- ☐ Wrong AMOUNT was given (mg or ml)
- ☐ Wrong FORMULATION and AMOUNT
- ☐ Wrong ROUTE was used (Subcutaneous,IM, IV)
- ☐ BOLUS was given instead of INFUSION
- ☐ Other

Please briefly explain how the incorrect dosing occurred.

Have you cared for any additional patients who received an INCORRECT DOSE of epinephrine administered in the ED for an allergic reaction or anaphylaxis?

- ☐ Yes
- ☐ No

For the second patient, with which formulation of epinephrine did the INCORRECT DOSING occur?

- ☐ EpiPen or EpiPen Jr
- ☐ IM epinephrine with syringe
- ☐ Subcutaneous epinephrine
- ☐ BOLUS IV epinephrine
- ☐ IV epinephrine INFUSION
- ☐ Unsure

For the second patient who received the incorrect dose of epinephrine, how did the incorrect dosing occur?

- ☐ Wrong FORMULATION used (1:1000 instead of 1:10,000 or vice versa)
- ☐ Wrong AMOUNT was given (mg or ml)
- ☐ Wrong FORMULATION and AMOUNT
- ☐ Wrong ROUTE was used (Subcutaneous,IM, IV)
- ☐ BOLUS was given instead of INFUSION
- ☐ Other

For the second patient, please briefly explain how the incorrect dosing occurred.

Please answer the following questions about yourself.

I am a:

- ☐ Nurse
- ☐ PA/NP
- ☐ Resident
- ☐ Consultant
- ☐ Pharmacist

Gender

- ☐ Female
- ☐ Male

Number of years in practice (since graduation from professional school.)

- ☐ 0-3
- ☐ 4-9
- ☐ 10-20
- ☐ >20

Do you have any comments regarding use of the EpiPen or Syringe/Ampule for epinephrine administration?
